# Antibiotic Resistance Profile of Bacteria Isolated from Wastewater Systems in Eastern Ethiopia

**DOI:** 10.1155/2020/2796365

**Published:** 2020-09-15

**Authors:** Awugchew Teshome, Tadesse Alemayehu, Wegene Deriba, Yohanes Ayele

**Affiliations:** ^1^Arba Minch University, College of Medicine and Health Sciences, Environmental Health Department, Arba Minch, Ethiopia; ^2^Haramaya University, College of Health and Medical Sciences, Environmental Health Department, Harer, Ethiopia; ^3^Hawassa University, College of Medical and Health Sciences, School of Pharmacy, Hawassa, Ethiopia

## Abstract

World Health Organizations launched a global action plan on antimicrobial resistance since 2015. Along with other objectives, the plan was aimed to strengthen knowledge of the spread of antimicrobial resistance through surveillance and research. Given their high bacterial densities and that they receive antibiotics, metals, and other selective agents, wastewater systems are a logical hotspot for antibiotic resistance surveillance. The current study reports on the result of antibiotic resistance surveillance conducted in selected wastewater systems of Eastern Ethiopia from Feb. 2018 to Oct. 2019. We monitored three wastewater systems in Eastern Ethiopia, such as the activated sludge system of Dire Dawa University, waste stabilization pond of Haramaya University, and a septic tank of Hiwot Fana Specialized University Hospital for 18 months period. We collected 66 wastewater samples from 11 sampling locations and isolated 722 bacteria using selective culture media and biochemical tests. We tested their antibiotic susceptibility using the standard Kirby-Bauer disk diffusion method on the surface of the Mueller-Hinton agar and interpreted the result according to EUCAST guidelines. The result shows the highest percentage of resistance for ampicillin among isolates of hospital wastewater effluent which is 36 (94.7%), 33 (91.7%), and 32 (88.9%) for *E. coli, E. faecalis*, and *E. faecium*, respectively. A lower rate of resistance was seen for gentamicin among isolates of activated sludge wastewater treatment system which is 10 (16.4%), 8 (13.3%), 11 (18.9%), and 12 (20.3%) for *E. coli, E. faecalis, E. faecium,* and *P. aeruginosa*, respectively. Hospital wastewater exhibited higher resistance than the other two wastewater systems. The Multiple Antibiotic Resistance Index (MARI) has significantly increased in the wastewater's course treatment process, showing the proliferation of resistance in the wastewater treatment system.

## 1. Introduction

Increased resistance of microorganisms to commonly prescribed antibiotics has become a major challenge in the current medical practice. Considering the threat it poses on global public health, the World Health Organization (WHO) declares that antibiotic resistance is a “major threat to public health” [1]. This increasing trend in antibiotic resistance would leave routine infections without effective treatment, and surgeries would become dangerous and rise healthcare practice expenditure [2].

Harboring a large number of commensal human and animal bacteria along with antibiotic resistance determinants, wastewater systems are antibiotic resistance hotspots, where antibiotic resistance develops, proliferates, and discharges into the environment [3–5]. However, data reported in previous publications are sometimes inconsistent and contradictory. For example, [6, 7] showed that due to the continuous exposure of bacteria to subinhibitory concentrations of antibiotics, wastewater treatment plants provide an environment that is potentially suitable for the proliferation of antibiotic resistance genes (ARGs) and antibiotic-resistant bacteria (ARBs). On the contrary, [8] showed that continuous exposure of a triclosan-sensitive *Staphylococcus aureus* strain to subinhibitory concentrations of triclosan did not promote any changes in triclosan susceptibility or other targeted antibiotics.

Despite the efforts to elucidate the role of wastewater treatment plants (WWTPs) in relation to antibiotic resistance, there is still no clear evidence that WWTPs, especially the biological treatment processes, are contributing to the proliferation of antibiotic resistance. Some studies suggest that WWTPs achieve a significant reduction in the number of ARBs [9, 10], while other studies indicate that WWTPs serve as major contributors of ARBs and ARGs [11]. These uncertainties may arise from research evaluating different treatment technologies, operational conditions, influent wastewater quality or wastewater constituents, and different methodologies for the detection of ARBs and ARGs. Therefore, additional studies and analyses are needed to assess the role of wastewater treatment processes on the proliferation and mitigation of antibiotic resistance. Hence, this study was conducted to assess the resistance pattern of environmental resistance indicator and examine the prepotency of wastewater systems to intensify antibiotic resistance of indicator organisms.

## 2. Method

### 2.1. Study Setting and Sampling Locations

**Figure 1:**
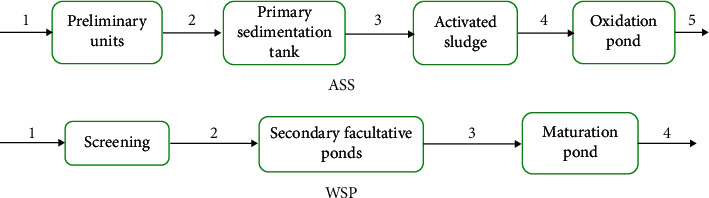
Schematic diagram of unit operations and unit processes and sampling locations. (a) Activated sludge system at Dire Dawa University with the five sampling locations. (b) Waste stabilization pond at Haramaya University with the four sampling locations.

Antibiotic resistance monitoring was conducted at selected wastewater systems (activated sludge system, waste stabilization pond, and septic tank system) in Eastern Ethiopia from October 2018 to April 2019. The activated sludge system and waste stabilization pond were full-scale plants receiving sewage from dormitories, cafeteria, animal farms, and laboratories at Dire Dawa University and Haramaya University, respectively (Figure 1). The third monitoring site is the septic tank system receiving hospital wastewater at Hiwot Fana Specialized University Hospital (HFSUH). The Dire Dawa University wastewater treatment plant is an activated sludge system (ASS) composed of preliminary waste treatment units (grit removal and stabilization basin), a primary sedimentation tank (Dortmund tank), an activated sludge system (aeration unit and secondary sedimentation), and waste oxidation pond. Haramaya University wastewater treatment plant is a waste stabilization pond (WSP) composed of a screening unit, two primary facultative ponds, and one maturation pond. Wastewater samples were collected at influent and effluent locations at each unit operation/process in the course of wastewater treatment.

### 2.2. Sample Collection

Wastewater samples were collected on a quarterly basis in October 2018–April 2019 from the specified sampling locations in the wastewater system. Plastic containers sterilized with 70% (v/v) alcohol were used to collect samples. During sampling, sample containers were rinsed three times with sample water before filling with the sample. To obtain a flow representative sample, the actual samples were obtained by integrating grab samples collected in a 30-minute interval in the morning hours at 8–11 am. After collection, the samples were protected from direct sunlight and transported in a cooler box containing ice packs to the laboratory for analyses. All samples were stored at 4^◦^C and analyzed within 24 h of sample collection.

### 2.3. Wastewater Characteristics

Wastewater samples were analyzed for pH on-site using a digital pH meter. Chemical Oxygen Demand (COD), Biochemical Oxygen Demand (BOD), and total suspended solid were analyzed in the laboratory according to standard methods [12]. Data on operational conditions such as flow rate, residence time, and desludging rate of wastewater treatment plant were collected each time of the wastewater sampling.

### 2.4. Bacterial Enumeration, Isolation, and Identification

Water samples were analyzed for the target bacterial using standard methods for the examination of water and wastewater [12]. Samples were thoroughly mixed to distribute the bacteria uniformly prior to analysis. Serial dilutions (10-2-10-6) of samples were prepared in sterile distilled water. Fifty milliliters from replicates of dilution of each sample was filtered using a 0.45 *µ*m, 47 mm, diameter, cellulosic white grid filter placed on the filter holder. Approximately 25 ml of distilled water was first added to wet the filter paper. Media were selected according to the procedure recommended by the manufacturer and sterilized by autoclaving at 15 lbs pressure (121°C) for 15 minutes. Membrane filters were aseptically transferred to 45 mm Petri dishes with the appropriate selective media.

R2A agar was used for the enumeration of total heterotrophic bacteria after incubation at 37°C for 24 hours. mEndo-LES agar was used for total coliform and mFC agar for the fecal coliform count after incubation at 37°C and 44.5°C for 24 hours, respectively. m-TEC agar was used for the enumeration of Thermotolerant *E. coli* 35–37°C for 2 hours and at 44.5 ± 0.5°C for 22 hours. For the isolation of *Enterococcus faecalis* and *Enterococcus faecium,* m-Enterococcus agar was used. Plates were incubated at 37°C, and results were read after 24 and 48 h. A maximum of five randomly selected presumptive *Enterococcus* colonies from mEnterococcus agar were subcultured on *Enterococcus* Differential Agar Base (TITG Agar Base) for the differentiation between *Enterococcus faecalis* and *Enterococcus faecium*. After incubation at 35–37°C for 18–24 hours with 1% TTC solution, colonies with a deep red center and a narrow white periphery were identified as *Enterococcus faecalis*, whereas white or pale pink colored colonies were identified as *Enterococcus faecium*. Cetrimide Agar was used for the isolation of *Pseudomonas aeruginosa* after incubation at 37°C for 48 h. We used *mADA*-V agar for the isolation of *Aeromonas spp*. incubated in a temperature-controlled incubator at incubation conditions shown in Table 1. The plates were labeled with a wastewater treatment plant, sampling location, date, and sample number.

### 2.5. Antimicrobial Susceptibility Test

Two isolates per sample of each bacterial species were collected to perform antimicrobial susceptibility testing (AST) except for hospital wastewater, for which three isolates were collected. The standard Kirby-Bauer disk diffusion method was used to determine the antimicrobial susceptibility profiles of the isolates [13]. Bacterial inoculums were prepared by suspending the freshly grown bacteria in 4–5 ml normal saline, and the turbidity was adjusted to that of a 0.5 McFarland standard. Then, this suspension was spread on over the entire surface of the Mueller-Hinton agar using a cotton swab to produce confluent growth.

The susceptibility test was performed by placing paper disks impregnated with specific amounts of antibiotics on a lawn of bacteria grown on agar and aerobically incubated at 35 + 1°C for 18–24 hours. After an incubation period, the diameter for the zone of inhibition, the area around the disk without bacterial growth, was measured.

Phenotypic resistance is often interpreted based on clinical standards and recommended breakpoints. A more reliable alternative for the interpretation of the antibiotic resistance of environmental bacteria may be the epidemiological cut-off (ECOFF) value developed by the European Committee on Antimicrobial Susceptibility Testing (EUCAST), which, in a given taxonomic group, separates the populations with acquired resistance mechanisms (non-wild-type) from the wild-type populations that have no resistance. In contrast to clinical breakpoints, the ECOFF values are epidemiologically based, do not relate to the therapeutic efficiency, and do not differ among different committees [14]. The inhibition zone diameters for this study were interpreted according to EUCAST guidelines [15], except *E. coli* tested for tetracycline, *Enterococci* tested which were evaluated by the Clinical Laboratory Standards Institute [16] guidelines. Disk content and breakpoints for each antibiotic used in this study are shown in Table 2.

### 2.6. Multiple Antibiotic Resistance Index (MARI)

MARI was determined for each isolate by using the formula MARI=*a*/*b*, where *a* represents the number of antibiotics to which the test isolate depicted resistance and *b* represents the total number of antibiotics to which the test isolate has been evaluated for susceptibility [17]. A MARI value of 0.2 indicates a high-risk environment where antibiotics are often used [18, 19].

### 2.7. Analysis

Data analysis was done using descriptive and inferential statistical tools in the R programming environment. A *P* value of ≤0.05 was considered a statistically significant difference. Box plot graphs were chosen to illustrate the distribution of the MERI values using the mean values. In order to decide which statistical test should be used for determining the significance, the data were first analyzed for their normal distribution using the Shapiro-Wilk test. The data were not normally distributed, and the Kruskal–Wallis test, a nonparametric version of the classical one-way analysis of variance (ANOVA), was used to determine variations in the level of antibiotic resistance (as measured by MERI) among studied bacterial groups. The result was used to assert whether antibiotic resistance level is significantly different among the three monitored systems and antibiotic resistance level varies in the course of wastewater treatment progress.

## 3. Result and Discussion

In the specified monitoring period, 66 samples were collected from 11 sampling locations in the three monitoring sites in six monitoring rounds sampled quarterly from Feb. 2018 to Oct. 2019 (Table 3). A total of 722 bacterial isolates proposed to indicate the level of antibiotic resistance in the monitored wastewater systems were isolated and analyzed for their susceptibility to commonly prescribed antibiotics.

### 3.1. Physicochemical and Bacteriologic Characteristics of Wastewater

Selected physicochemical and biological characteristics of wastewater analyzed were presented in Table 4. Wastewater characteristics (both physicochemical and microbial load) of the three systems were almost comparable. However, animal farm waste entering Haramaya University waste stabilization pond was the strongest waste in both organic and bacterial load with mean BOD and COD measure of 1108.33 and 1275.33 mg/L, respectively, and with 5.55 *∗* 10^8^, 2.74 *∗* 10^8^, and 1.13 *∗* 10^8^ cfu/100 mL for *total coliform*, *fecal coliform*, *Enterococci.* spp., and *E. coli,* respectively. The pH of effluent from the maturation pond in the waste stabilization pond of Haramaya University and the oxidation pond of the activated sludge system of Dire Dawa University was 9.25 and 9.45, respectively. The treatment efficiency of wastewater plants was presented in Table 4 as log reduction for the specific physicochemical and bacterial contaminants. The efficiency of removal at log scale ranges from 0.83 for COD removal at WSP to 3.21 for total coliform at activated sludge system.

BOD to COD ratio is an important aggregate measure of wastewater characteristics, indicating the biodegradability of wastewater [20] and microbial community [21, 22]. Typical values for the ratio of BOD/COD for untreated domestic wastewater are in the range from 0.3 to 0.8 [23]. BOD/COD ratio of the waste treated in ASS and WSP is in the range from 0.79 of raw wastewater to 0.61 of effluent wastewater and from 0.87 of raw wastewater to 0.46 of effluent wastewater, respectively. This makes it suitable for biological treatment, which makes ASS and WSP the right choice for the treatment of such waste. A high level of efficiency of microbial removal was achieved ranging from 95% to 99% at both ASS and WSP. This may be related to the interplay between sunlight, algal growth, and elevated pH [24].

### 3.2. Antimicrobial Susceptibility Profile of Bacterial Isolates

**Figure 2:**
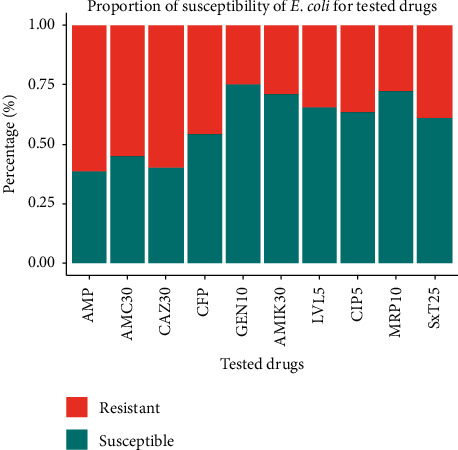
Level of antibiotic susceptibility among *E. coli* isolated from the three monitored sites.

This study evaluated ten commonly prescribed antibiotics against five groups of bacteria proposed to indicate antibiotic resistance level in the environment and the results are presented in Table 5. From the three monitored sites and across the course of wastewater treatment, 151 *E. coli* were isolated and tested for their resistance pattern against ten commonly prescribed antibiotics. The antibiotic resistance pattern of *E. coli* is presented in Figure 2. As shown, *E. coli* resistance is higher for *β*-Lactams and Cephalosporin groups such as ampicillin and amoxicillin/clavulanic acid while it is lower for Aminoglycosides (Gentamicin and Amikacin), and carbapenem (Meropenem) groups.

Isolates have shown reduced susceptibility for *β*-Lactams and Cephalosporin (Ampicillin, Amoxicillin/Clav, and Ceftazidime) across all monitoring locations. Similarly, across all sites, isolates have shown higher susceptibility to Aminoglycosides (Gentamicin and Amikacin) and carbapenem (Meropenem). The highest frequency of resistance was recorded against ampicillin 94.7% for *E. coli* isolates from hospital wastewater, followed by ceftazidime with a frequency of 86.8% for *E. coli* isolates from hospital wastewater. Except for ampicillin, amoxicillin/clav, and ceftazidime, the resistance frequencies displayed by the isolates against other antibiotics were <50%, as shown in Figure 2.

**Figure 3:**
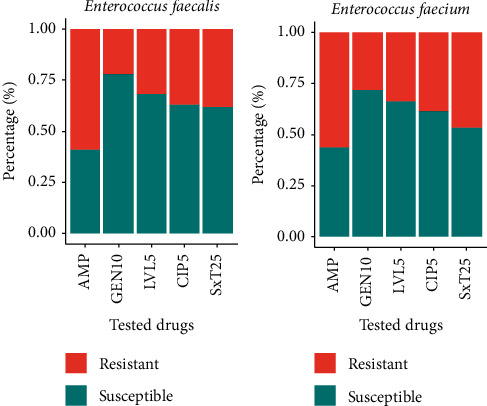
Level of antibiotic susceptibility among *Enterococcus* spp. isolated from the three monitored sites.

From the three monitored sites, a total of 286 *Enterococcus* spp. (144 *E. faecalis* and 142 *E. faecium*) were isolated, and their antibiotic resistance was tested against five commonly prescribed antibiotics such as ampicillin, gentamicin, levofloxacin, ciprofloxacin, and Co-Trimoxazole. The result of the antibiotic susceptibility test was presented in Figure 3. Both isolates of *Enterococcus* spp. exhibit a higher level of resistance for ampicillin and Co-Trimoxazole, while exhibiting higher susceptibility for gentamicin. The resistance of *E. faecalis* ranges from 13.3% for gentamicin in ASS wastewater to 91.7% for ampicillin in STS of hospital wastewater. Similarly, antibiotic resistance of *E. faecium* is in the range from 18.9% for gentamicin in ASS wastewater to 88.9% for ampicillin in STS of hospital wastewater.

**Figure 4:**
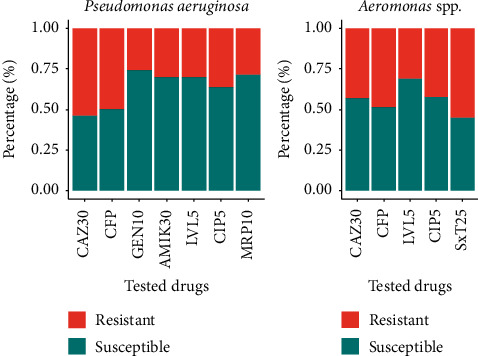
Level of antibiotic susceptibility among *P. aeruginosa* and *Aeromonas* spp. isolated from the three monitored sites.

A total of 143 *Pseudomonas aeruginosa* were isolated from ASS (59), WSP (48), and STS (36). The isolates were tested for antibiotic resistance activity against seven antibiotics, namely, ceftazidime, cefepime, gentamicin, amikacin, levofloxacin, ciprofloxacin, and Meropenem, and the result is presented in Figure 4. As shown, *Pseudomonas aeruginosa* isolates expressed a higher level of resistance for Cephalosporin such as ceftazidime and cefepime while showing higher susceptibility for gentamicin and meropenem. The resistance level is in the range from 18% resistance to meropenem among isolates of ASS to 77.8% resistance to ceftazidime and cefepime among isolates of hospital STS.

Form 66 samples collected from the three sites, 142 *Aeromonas* spp. were isolated and their antibiotic resistance profile was tested against five comment antibiotics such as ceftazidime, cefepime, levofloxacin, ciprofloxacin, and Co-Trimoxazole. Isolates expressed a higher level of antibiotic resistance Co-Trimoxazole, ceftazidime, and cefepime while expressing higher susceptibility for levofloxacin.

Based on the result of the susceptibility test shown in Table 5, isolates from hospital wastewater have shown elevated resistance characteristics for all isolates and drugs tested. The highest percentage resistance across all isolates was for AMP resistance for hospital wastewater which is 36 (94.7%), 33 (91.7%), and 32 (88.9%) for *E. coli*, *E. faecalis,* and *E. faecium,* respectively. Lower rate of resistance was seen for GEN10 for activated sludge wastewater treatment system which is 10 (16.4%), 8 (13.3%), 11 (18.9%), and 12 (20.3%) for *E. coli, E. faecalis, E. faecium, and P. aeruginosa,* respectively.

Accordingly, isolates from hospital wastewater have shown elevated resistance characteristics for all isolates and drugs tested while isolates of ASS expressed lower resistance for all isolates and tested drugs. The highest percentage resistance across all isolates was for ampicillin resistance for hospital wastewater which is 36 (94.7), 33 (91.7), and 32 (88.9) for *E. coli*, *E. faecalis*, and *E. faecium,* respectively. Lower rate of resistance was seen for gentamicin for activated sludge wastewater treatment system which is 10 (16.4), 8 (13.3), 11 (18.9), and 12 (20.3) for *E. coli, E. faecalis, E. faecium*, and *P. aeruginosa,* respectively. A higher level of *E. coli* resistance was seen in hospital wastewater which is in the range between 42.1% for meropenem and 94.7% for ampicillin.

The rate of isolation of resistant bacteria in the hospital wastewater was higher than that in the nonhospital environment for all indicator variables; this was statistically significant (*P* < 0.001). A similar observation was reported by [25]. The difference in the environmental resistance between the three wastewater systems may be explained by different types of source wastewater. Influent wastewater to ASS is dominated by human waste which comprises both antibiotic-resistant bacteria and antibiotic residues, a mixture that under favorable conditions, of high nutrient content and close contact between bacteria, may promote antibiotic resistance dissemination [26]. However, influent wastewater to WSP is dominated by animal husbandry wastewater, which may comprise a large amount of antibiotics' residue, which in turn contributes to elevated rate isolation of antibiotic resistance bacteria [27]. Factors other than the indiscriminate use of antibiotics in human medicine, animal husbandry, and agriculture may disrupt the microbial balance in favor of resistant bacteria. Hospitals are known to discharge pathogenic bacteria, most of which could be carrying resistance determinants into their wastewater, and traces of antibiotics in urine, feces, and spilled and expired drugs, which are improperly discarded into washbasins, are all channeled to the wastewater systems.

### 3.3. Change in Multidrug Resistance Level in Course of Wastewater Treatment

**Figure 5:**
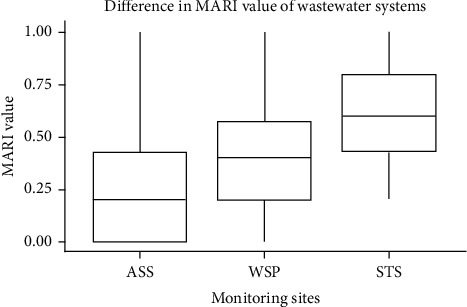
Box plot of mean MARI value in the three wastewater systems.

**Figure 6:**
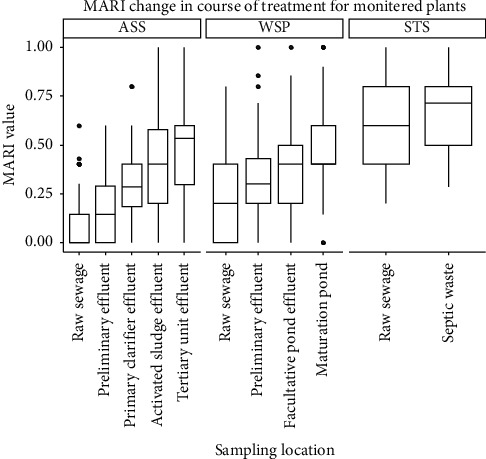
Change in MARI value in the course of wastewater treatment in the three studied sites.

The difference in antibiotic resistance level among the three monitored sites and its change in the course of the wastewater treatment process was shown in box plots of Figures 5 and 6, respectively. As shown in the box plot (Figure 5), there is a clear variation in the MERI value at three monitored sites with a higher rate of being multidrug resistance in STS of the hospital wastewater.

Multidrug resistance level as measured by the mean value of MERI has also shown clear variation at each stage in the three monitored sites. The bar graph in Figure 6 depicted a change in the mean MERI value in the course of the wastewater treatment process. For each of the wastewater sites monitored, effluent has a higher level of MERI compared with raw wastewater. An increase has also been shown at each stage for ASS and WSP.

MARI has been used to estimate the health risks associated with the spread of drug resistance in an environment. A MARI value of 0.2 (arbitrary) is used to differentiate between low and high health risks, and MARI greater than 0.2 suggests that strain(s) of bacteria originate from an environment with high contamination or antibiotics usage [18]. The MARI estimates obtained for isolates from our study sites were 0.287, 0.36, and 0.639, for ASS, WSP, and STS, respectively. These measures were all greater than 0.2, suggesting that the isolates originated from environments with high use or contamination of antibiotics. The high MARI values obtained in this study may suggest the exposure of the isolates to antibiotics pressure, which might have resulted from inappropriate use of antibiotics among the population in the study area, and may lead further to an increase in the development of multidrug resistance overtime if appropriate measures are not put in place [28].

This study showed that the intensity of resistance increases in the course of the wastewater treatment process. There is a clear increase in the measure of multidrug resistance profile of isolates in the course of the wastewater treatment process. This can be taken as an indication of the propensity of wastewater systems to intensify antibiotic resistance. Currently, there is no clear evidence of whether resistance may develop in wastewater treatment plants (WWTPs) [29]. The cause-effect relationship has not yet been well established between the presence of antibiotic resistance determinants in the wastewater treatment plant and the favoring of resistant bacteria. However, there is established evidence that wastewater, or even treated wastewater, contains higher proportions of various resistant bacteria populations in relation to the respective proportions contained in other aquatic environments [10]. As per former studies, the conditions in wastewater treatment plants are favorable for the proliferation of ARB and nonresistant bacteria to acquire resistance genes [30]. Goñi-Urriza et al. [31] monitored the population of antibiotic-resistant bacteria in the effluent of the wastewater treatment plant and receiving river, and in the antibiotic susceptibility tests, it was found that resistance against 21 out of the 22 antibiotics tested was significantly increased among the strains of *Enterobacteriaceae* and *Aeromonas* spp. collected downstream of the wastewater discharge point. Iwane et al. [32] also reported that the ratio of tetracycline-resistant coliforms increased by up to 6.8% downstream of a wastewater treatment plant.

This report has numerous strengths; to mention some, we have tried to avoid the wrong “high resistance” alarm by excluding bacteria/antibiotic combination that leads to intrinsic resistance. We have significantly reduced redundant testing in the case where cross-resistance is a rule. Although this study addresses important environmental health issues, it is not free from limitations. We are unable to identify the genes responsible for expressed resistance. We are also unable to determine the level of antibiotic resistance determinants such as antibiotic residue, heavy metal concentration, and antibiotic resistance genes in the wastewater systems. In addition, carbapenemase and extended-spectrum beta-lactamase pattern of isolated bacterial species were not determined.

## 4. Conclusions

Our antibiotic resistance surveillance program has shown the role of wastewater systems in the proliferation of antibiotic resistance in the wastewater systems. The study has found a high level of environmental antibiotic resistance indicator bacteria thrive in the wastewater systems. Multiresistance patterns to antibiotics were common among the isolates. The percentages of resistance in the wastewater treatment plant were increased through the course of treatment. Hospital wastewater exhibited higher resistance to tested antibiotics than the other two wastewater systems. The multidrug resistance index has significantly increased in the advancement of the wastewater treatment process for all wastewater treatment plants. This may indicate the proliferation of resistance in the wastewater treatment system.

The presence of antibiotic-resistant organisms in these wastewater systems should not be overlooked. For the future, wastewater systems should be designed to control the dissemination of antibiotic-resistant bacteria. Further disinfection or other advanced treatment processes have to be included in the treatment design. It is also imperative that wastewater discharge compliance monitoring should determine antibiotic susceptibility/resistance patterns of isolated microbes beyond traditional efficiency measures. Further studies should be conducted in the region to determine antibiotic-resistant determinants in the wastewater system such as antibiotic residue and resistance genes.

## Figures and Tables

**Table 1:** Media and incubation conditions used for the enumeration, and primary isolation of the indicated bacteria from wastewater samples.

Bacteria	Media	Incubation conditions
Total heterotrophic count	R2A agar	37°C; 24 h
Total coliforms	mEndo-LES agar	37°C; 24 h
Fecal coliforms	mFC agar	44.5°C; 24 h
*Enterococcus spp*. (*E. faecalis and E. faecium*)	mEnterococcus agar + TITG agar base	37°C; 48 h ← 35–37°C for 18–24 hours
*Escherichia coli*	m-TEC agar	35–37°C for 2 hours and at 44.5 ± 0.5°C for 22 hours
*Aeromonas* spp.	mADA-V agar	37°C; 24 h
*Pseudomonas aeruginosa*	Cetrimide agar	35°C for 18 h

**Table 2:** Disk content and EUCAST breaking points of each antibiotic tested for specific indicator bacteria.

Antibiotic class	Antibiotic	Code	Content (*µ*g)	*E. coli*		*Enterococci* spp.		*P. aeruginosa*		*Aeromonas* spp.	
				<R	≥S	<R	≥S	<R	≥S	R	S
*β*-lactams	Ampicillin^*∗*^	AMP	10|2	14	14	8	10	IR		NR	
	Amoxicillin/Clav	AMC30	20/10	19	19			IR		NR	
Cephalosporin	Ceftazidime	CAZ30	10	19	22	IR		17	17	21	24
	Cefepime	CFP	30	24	27	IR		21	21	24	27
Aminoglycosides	Gentamicin^*∗*^	GEN10	10|30	14	17	8	8	15	15	NR	
	Amikacin	AMIK	30	15	18			15	18	NR	
Fluoroquinolone	Levofloxacin	LVL5	5	23	19	15	15	22	22	24	27
	Ciprofloxacin	CIP5	5	24	26	15	15	26	26	24	27
Carbapenem	Meropenem	MRP10	10	16	22	NR		18	24		
Sulfonamides	Co-Trimoxazole	SxT25	1.25/23.75	11	14	23	23	NR		16	19

IR: intrinsically resistant, NR: not recommended. ^*∗*^We have used two types of ampicillin and gentamicin disk for AST of *E. coli and Enterococci* spp.

**Table 3:** Number of samples and bacterial isolates obtained per monitoring sites.

Site	No. of sampling points	No. of sample	Number of isolate				
			*E. coli*	*E. faecalis*	E. faecium	*P. aeruginosa*	*Aeromonas* spp.
ASS	5	30	61	60	58	59	58
WSP	4	24	52	48	48	48	48
STS	2	12	38	36	36	36	36
Total	11	66	151	144	142	143	142

ASS: activated sludge system, WSP: waste stabilization pond, STS: septic tank system.

**Table 4:** Mean value of selected biological and physicochemical characteristics of raw and effluent wastewater in the three monitoring sites and removal capacity of wastewater treatment facilities.

Site	Characteristics	Total coliform cfu/100 ml	Fecal coliform cfu/100 ml	*Enterococci* spp. cfu/100 ml	*E. coli* cfu/100 ml	BOD mg/L	COD mg/L	TSS mg/L	pH
ASS	Raw	5.14 *∗* 10^8^	2.45 *∗* 10^7^	1.31 *∗* 10^8^	1.17 *∗* 10^7^	737.67	931.33	707.67	7.23
	Effluent	3.18 *∗* 10^5^	5.12 *∗* 10^4^	3.93 *∗* 10^5^	2.75 *∗* 10^4^	73.17	119.33	63.50	9.45
	Log reduction	3.21	2.68	2.52	2.62	1.003	0.89	1.04	

WSP	Raw	5.55 *∗* 10^8^	2.74 *∗* 10^8^	1.13 *∗* 10^8^	9.87 *∗* 10^6^	1108.33	1275.33	951.67	7.45
	Effluent	9.9 *∗* 10^6^	7.28 *∗* 10^5^	3.33 *∗* 10^5^	5.36 *∗* 10^4^	91.17	187.67	60.67	9.25
	Log reduction	1.74	2.57	2.53	2.26	1.08	0.83	1.19	

STS	Raw	1.39 *∗* 10^8^	7.93 *∗* 10^7^	9.3 *∗* 10^7^	1.53 *∗* 10^7^				
	Effluent	3.5 *∗* 10^6^	3.9 *∗* 10^6^	4.28 *∗* 10^6^	4.06 *∗* 10^5^				
	Log reduction	1.59	1.3	1.33	1.57				

ASS: activated sludge system, WSP: waste stabilization pond, STS: septic tank system, BOD; biochemical oxygen demand, COD: chemical oxygen demand, TSS: total suspended solid.

**Table 5:** Antibiotic resistance among isolates of environmental resistance indicator bacterial species by monitoring site.

Site	Resistance phenotype	No. tested	Number and (%) resistant to antibiotic tested										MARI mean (SD)
			AMP	AMC30	CAZ30	CFP	GEN10	AMIK	LVL5	CIP5	MRP10	SxT25	
ASS	*E. coli*	61	29 (47.5)	28 (45.9)	30 (49.2)	19 (31.2)	10 (16.4)	13 (21.3)	14 (22.9)	17 (27.87)	11 (18)	15 (24.6)	0.30 (0.03)
	*E. faecalis*	60	26 (43.3)	–	–	–	8 (13.3)	–	13 (21.6)	15 (25)	–	16 (26.7)	0.26 (0.03)
	*E. faecium*	58	25 (43.1)	–	–	–	11 (18.9)	–	12 (20.7)	15 (25.9)	–	18 (31)	0.28 (0.04)
	*P. aeruginosa*	59	–	–	25 (42.37)	23 (39)	12 (20.3)	12 (20.3)	12 (20.3)	16 (27.1)	11 (18.6)	–	0.27 (0.03)
	*Aeromonas* spp.	58	–	–	19 (32.8)	21 (36.2)	–	–	12 (20.7)	17 (29.31)	–	24 (41.4)	0.32 (0.04)

WSP	*E. coli*	52	28 (53.8)	25 (48.1)	27 (51.9)	19 (36.5)	13 (25)	14 (26.9)	17 (32.7)	19 (36.54)	15 (28.9)	15 (28.9)	0.37 (0.03)
	*E. faecalis*	48	26 (54.2)	–	–		11 (22.9)	–	14 (29.2)	17 (35.4)	–	17 (35.4)	0.35 (0.03)
	*E. faecium*	48	23 (47.9)	–	–		11 (22.9)	–	13 (27.1)	17 (35.4)	–	21 (43.7)	0.35 (0.03)
	*P. aeruginosa*	48	–	–	24 (50)	20 (41.7)	9 (18.75)	48 (29.2)	14 (29.2)	15 (31.3)	13 (27.1)	–	0.32 (0.04)
	*Aeromonas* spp.	48	–	–	16 (33.3)	19 (39.6)	–	–	13 (27.1)	18 (37.5)	–	29 (60.4)	0.40 (0.04)

STS	*E. coli*	38	36 (94.7)	30 (78.9)	33 (86.8)	31 (81.6)	15 (39.5)	17 (44.7)	21 (55.26)	19 (50)	16 (42.1)	29 (76.32)	0.65 (0.03)
	*E. faecalis*	36	33 (91.7)	–	–		13 (36.1)	–	19 (52.8)	21 (58.3)	–	22 (61.1)	0.60 (0.04)
	*E. faecium*	36	32 (88.9)	–	–		18 (50)	–	23 (63.9)	23 (63.9)	–	27 (75)	0.68 (0.04)
	*P. aeruginosa*	36	–	–	28 (77.8)	28 (77.8)	16 (44.4)	17 (47.2)	17 (47.22)	21 (58.3)	17 (47.2)	–	0.57 (0.03)
	*Aeromonas* spp.	36	–	–	26 (72.2)	29 (80.6)	–	–	19 (52.8)	25 (69.4)	–	25 (69.4)	0.69 (0.4)

ASS: activated sludge system, WSP: waste stabilization pond, STS: septic tank system, AMP: ampicillin, AMC30: amoxicillin/clav, CAZ30: ceftazidime, CFP, cefepime, GEN10: gentamicin, AMIK: amikacin, LVL5: levofloxacin, CIP5: ciprofloxacin, MRP10: meropenem, SxT25: Co-Trimoxazole.

## Data Availability

The data used to support the findings of this study are available from the corresponding author upon request.
